# New strategy for automatic tumor segmentation by adaptive thresholding on PET/CT images

**DOI:** 10.1120/jacmp.v13i5.3875

**Published:** 2012-09-06

**Authors:** Mazen Moussallem, Pierre‐Jean Valette, Alexandra Traverse‐Glehen, Claire Houzard, Christophe Jegou, Francesco Giammarile

**Affiliations:** ^1^ Nuclear Medicine Unit Imaging Department Centre Hospitalier Lyon‐Sud Pierre‐Bénite; ^2^ Anatomo‐pathology Department Centre Hospitalier Lyon‐Sud Pierre‐Bénite; ^3^ Radiology Unit Imaging Department Centre Hospitalier Lyon‐Sud Pierre‐Bénite; ^4^ EMR 3738 Faculté de Médecine Lyon‐Sud Université Claude Bernard Lyon 1 France

**Keywords:** adaptive thresholding, FDG‐PET/CT, automatic segmentation, radiotherapy oncology

## Abstract

Tumor delineation is a critical aspect in radiotherapy treatment planning and is usually performed with the anatomical images of a computed tomography (CT) scan. For non‐small cell lung cancer, it has been recommended to use functional positron emission tomography (PET) images to take into account the biological target characteristics. However, today, there is no satisfactory segmentation technique for PET images in clinical applications. In the present study, a solution to this problem is proposed. The development of the segmentation technique is based on the threshold's adjustment directly from patients, rather than from phantoms. To this end, two references were chosen: measurements performed on CT images of the selected lesions, and histological measurements of surgically removed tumors. The inclusion and exclusion criteria were chosen to produce references that are assumed to have measured tumor sizes equal to the true *in vivo* tumor sizes. In total, for the two references, 65 lung lesions of 54 patients referred for FDG‐PET/CT exams were selected. For validation, measurements of segmented lesions on PET images using this technique were also compared to CT and histological measurements. For lesions greater than 20 mm, our segmentation technique showed a good estimation of histological measurements (mean difference between measured and calculated data equal to −0.8±9.0%) and an acceptable estimation of CT measurements. For lesions smaller than or equal to 20 mm, the method showed disagreement with the measurements derived from histological or CT data. This novel segmentation technique shows high accuracy for the lesions with largest axes between 2 and 4.5 cm. However, it does not correctly evaluate smaller lesions, likely due to the partial volume effect and/or respiratory motions.

PACS numbers: 87.53.Bn, 87.53.Kn, 87.55.D, *87.57.nm*, 87.57.U

## I. INTRODUCTION

The aim of radiotherapy is to deliver the highest possible dose of ionizing radiation in the target tumor volume while minimizing the irradiation of surrounding healthy tissue. Tumor underdosing causes a recurrence, whereas irradiation of surrounding healthy tissue can cause serious side effects. Therefore, it is very important to precisely determine the tumor limitations.

It has been shown that the definition of target volumes, with anatomical images such as computed tomography (CT), represents one of the largest sources of error in radiotherapy treatment planning.^(1)^ Positron emission tomography (PET) with fluorine‐18‐fluorodeoxyglucose (^18^F‐FDG) can provide additional functional information on tumor volume.^2^, ^3^ For non‐small cell lung cancer, it has been recommended to use functional PET images to take into account the biological target characteristics.^4^, ^6^ Thus, the exact delineation of lung tumors on FDG‐PET images is a keystone in radiotherapy strategy.^7^, ^8^ However, for lung cancers, PET images are affected by poor spatial resolution, respiratory movements, and the tumor's heterogeneity.^9^, ^10^

Several segmentation methods have been proposed for tumor delineation on PET images, but there is no consensus in the community about an appropriate method that automatically defines a tumor volume on PET images. The main reasons are the lack of precision in real size tumor estimation, automatization, and limited quality of most methods.^(11)^

Among these segmentation methods, adaptive thresholding techniques^12^, ^16^ characterize each image by specific parameters such as background, maximum intensity of grayscale pixels, and size of lesions.^9^, ^17^ After adjustment, a mathematical “threshold adjustment function” will give a threshold value for each image corresponding to these specific parameters. Generally, this adjustment is obtained by the use of a phantom to represent a patient body. However, these adaptive thresholds methods suffer from problems and inaccuracies when applied to real tumors instead of to phantoms. To resolve these problems, some authors have worked on improving and changing the phantom, in order to give it shape and characteristics that more closely match those of the patient.^(18)^ Nevertheless, these adaptive thresholds methods still have reliability problems in clinical applications likely due to the phantom's inability to accurately represent the human body.

Histological data are used only for validation and comparison of segmentation techniques.^8^, ^19^, ^22^ Furthermore, there is no study that adjusts the threshold function by using histological data. Thus, the aim of this study is mainly focused on segmentation optimization by adjusting the threshold functions directly from the information extracted from the patient's body (from CT and histological data). By adjusting the function according to the human body instead of to the phantom, the function becomes easier to apply to real patient bodies (by using, for application, exactly the same procedures used for adjustment), thus yielding more accurate results.

## II. MATERIALS AND METHODS

### A. FDG‐PET/CT imaging

FDG‐PET/CT scans were performed with a dedicated hybrid PET/CT (Gemini, Philips Medical Systems, Cleveland, OH). The patients' images were recorded after 60 min from intravenous administration (5 MBq/kg) of FDG. The acquisition time was 3 min for each bed position. PET images were acquired with a field of view of 576 mm and a clinical spatial resolution of 7 mm. The CT images were obtained during quiet breathing and with 5 mm of space between two successive cuts. PET images were reconstructed with an attenuation correction, according to the Phillips “RAMLA 3D” software.

### B. Histological examination of pulmonary resection specimens

After surgical removal and orientation, the pathologist examined the fresh specimen by palpation in order to estimate the shape, size, and major orientation axis of the lesion. The surgical specimen was then fixed in formalin by injection into the bronchial airways and/or transparietal injection to restore the lung volume. Injection was stopped when the specimen was saturated and formalin was being exuded outside the surgical specimen. After 24 to 48 hours, a careful macroscopic processing measurement was performed on the fixed specimen. The first slice was cut parallel to the estimated major axis of the lesion, or parallel to the transversal plane of the patient (in the case of a transversal major axis). All subsequent sections were parallel to the first at intervals of 3 mm. Then, for each slice of each lesion, the major axis was measured and the largest of the major axes was recorded. Finally, all lesion slices were counted then multiplied by 3 mm to deduce the lesion's axis size in the cut direction. The result was later checked against the recorded major axis. Therefore, only the major axis of each lesion was recorded.

### C. Inclusion and exclusion criteria

The inclusion and exclusion criteria were chosen to produce references that are assumed to have measured tumor sizes equal to the true *in vivo* tumor sizes.

Data were obtained from the FDG‐PET/CT exam database of our Nuclear Medicine Department, and analyzed from June 2007 to June 2010. Only suspected lung lesions presenting pathologic FDG uptake were considered. The selected lesions were all located in the upper lobes of the lungs (to limit the respiratory movement effects^23^, ^24^) and had diameters smaller than 4.5 cm.

Based on the reference used to determine the adjustment function in the automatic segmentation algorithm, patients were divided into two groups. The first group's reference is based on the CT images (CTG: CT Group) from PET/CT exams with lesions (solitary pulmonary nodule and/or isolated lung masses for lesions bigger than 3 cm in diameter) that present only homogeneous FDG uptake, and have a well‐defined border on CT images. This selection was made by the nuclear physicians. The second group's reference is based on the histological measurements of the surgically removed lesions. In this histological group (HG), lesions were selected only if the major axis measured on the histological specimen was oriented parallel to the transverse plane of the patient. This choice of the tumor's major axis in the patient's transverse plane was made so that it can later be compared with the length of the major axis that appears on a transverse PET image. Furthermore, the period between FDG‐PET/CT exam and surgery must not exceed one month.

Patients with pulmonary atelectasis (lung collapse), patients who have already been treated by radiotherapy or by chemotherapy, those with necrosis central lesions (tissue death), or those with lesions presenting rapid extension were excluded from this study.

### D. Determination of the “threshold adjustment function” for segmentation

#### D.1 First reference from CT images

In this first reference group, for each lesion, only one transversal section (only one slice) of CT and PET images was used to adjust the threshold function described later in this study. This transversal section of CT contains the largest area of lesion. A first step was to find the PET image corresponding to the selected CT image by fusion software. To automatically identify the lung region on the PET image, the corresponding CT image was superimposed. Subsequently, each lesion was manually segmented on the transversal section of the CT image by an expert with more than 15 years of experience in PET and CT. Then, the area (Area CT) and major axis (MaAxis CT) of each segment were recorded. For the corresponding PET image, several gray levels were used as an initial threshold (iTH). All of the iTH values that gave the closest lesion area on the PET image (Area PET) to the lesion area found on the corresponding CT image (Area CT) were retained and were called aTH (accepted threshold). The mean value between the maximum and the minimum of all aTHs was set as Threshold. Tmax and Tmean represent, respectively, the target (T) maximum and mean intensities (gray levels) forming Area PET. In order to obtain the Threshold as a percentage, the following ratio was used: %Tmean = [(Threshold/Tmean) x 100]. In addition, Bmin represents the minimum intensity measured in the two lungs on the PET image. Bmin is an empirical parameter and has no significant physical value. It has been chosen for its good relation with other parameters. All these parameters (Tmax, Tmean, and Bmin) are used to characterize and distinguish the PET images that correspond to different thresholds.

#### D.2 Second reference from surgical specimens

In this second reference group, only one PET transversal image of each lesion, giving the greatest area, was taken into account. To find the greatest area, all PET slices were first segmented using the “threshold adjustment function” adjusted from the CT image reference (from the previews section), and then checked by an expert (without knowledge of the first result). For different iTH values on the PET image, only the values that gave a major axis of the lesion (MaAxis PET) that was the closest to the major axis found by histological measurement on surgical specimens (MaAxis Hist) were accepted (aTH). Hence, the target parameter MaAxis PET can be obtained from different aTHs of PET images. The Threshold is the average between the maximum and minimum value of aTHs. In this case, Area PET is formed from the average of areas having MaAxis PET as a retained major axis on the segmented PET image. Tmean, Tmax, %Tmean, and Bmin parameters were determined in the same manner as shown in the previous section. Note that in this study, all data were analyzed and processed using “ImageJ” software.

#### D.3 Reference: final decision

For both references, the mathematical function of the trend curve (between the characteristic parameters of PET images and the corresponding thresholds used for lesion segmentation) was determined with six regression models: exponential, linear, logarithmic, polynomial, power, and moving average (from Microsoft Excel). The model that had the best (the biggest) coefficient value of determination of the regression (“$R^{2}$: R‐squared values”) was retained (because, as the coefficient value of determination rises, the associated function fits better in points). Note that parameters were chosen in a way to allow a good mathematical relationship with the threshold.

### E. Considered patients

In this study, 65 lung lesions from 54 patients were selected. Namely, 38 lesions (CTG: lesion #1 to lesion #38) from 27 patients were delimited on CT scans, and 27 lesions (HG: lesion #39 to lesion #65) from the other 27 patients were evaluated in the pathology department after being surgically removed.

## III. RESULTS

### A. Threshold adjustment function found using the CT images as a reference

**Figure 1:**
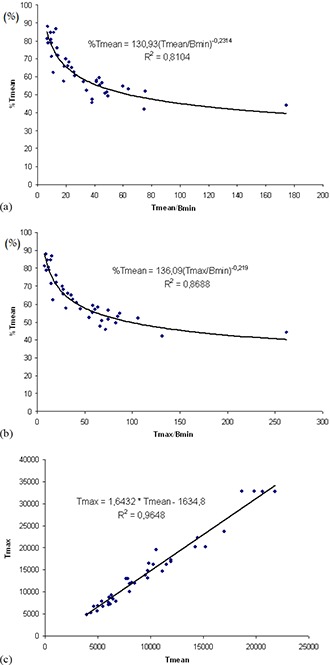
Reference (CT images): correlation between %Tmean and Tmean/Bmin (a), correlation between %Tmean and Tmax/Bmin (b), and correlation between Tmax and Tmean (c).

The points in (Fig. 1a) represent the values of %Tmean in terms of Tmean/Bmin, obtained from the first group. The mathematical function of the chosen regression curve, which represents the correlation between these parameters, is described below as function #1:

(1) $$\%\text{Tmean}=130.9\times {\left(\text{Tmean}/\text{Bmin}\right)}^{‐0.23}$$

with coefficient of determination $R^{2}=0.81$.

This function can be used as a “threshold adjustment function” for segmenting lesions on PET images that are characterized by the Tmean/Bmin parameters.

The points in (Fig. 1b) represent the values of %Tmean in terms of Tmax/Bmin, obtained from the CTG. The mathematical function of the chosen regression curve that represents the correlation between these parameters is described below as function #2:

(2) $$\%\text{Tmean}=136.09\times {\left(\text{Tmax}/\text{Bmin}\right)}^{‐0.22}$$

with coefficient of determination $R^{2}=0.87$.

The points in (Fig. 1c) represent the values of Tmax in terms of Tmean, obtained from the CTG. The mathematical function of the linear regression curve that represents the correlation between these parameters is named function #3 and is also described as follows:

(3) Tmax=1.64×Tmean‐1634.8

with a coefficient of determination $R^{2}=0.96$.

By combining functions #2 and #3, function #4 is obtained:

(4) $$\%\text{Tmean}=136.09\times {\left[1.64\times (\text{Tmean}/\text{Bmin})‐1634.8/\text{Bmin}\right]}^{‐0.22}$$

with a coefficient of determination $R^{2}=0.82$. (This coefficient of determination is calculated manually by generating a mathematical function on Microsoft Excel.)

**Figure 2:**
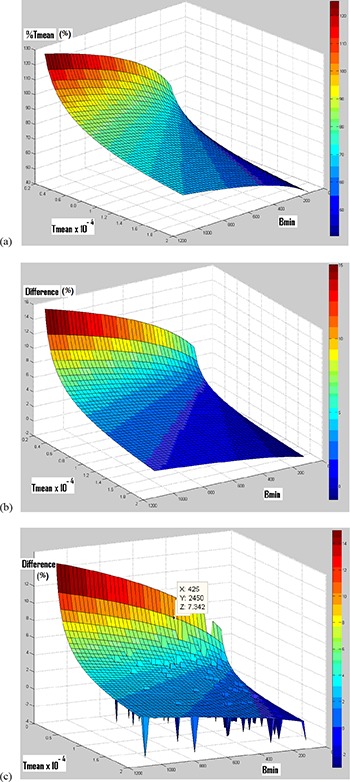
Function #4 in three dimensions (a); difference in percentage (%) between functions #4 and #8 (b); difference (in %) between functions #4 and #8, as well as the distribution of “clinical point” (c). (For a better visualization of the “clinical point” localization, their difference value is imposed to ‐3.)

Contrary to the next section, compared to function #1, function #4 is not significantly more accurate (due to its greater coefficient of determination), but it is considered the “threshold adjustment function”. This choice was selected because function #4 will be compared to another function in the next section, which is adjusted in the same way as function #4. Then function #4 will be later validated by being applied to the PET images of HG lesions (having histological measurements). (Figure 2a) shows function #4 in 3D. In this figure, %Tmean depends heavily on Tmean and lightly on Bmin.

### B. Threshold adjustment function found using histological measurements as a reference

Concerning the values of %Tmean in terms of Tmean/Bmin obtained from the second group, the mathematical function of the chosen regression curve that represents the correlation between these parameters is (function #5):

(5) $$\%\text{Tmean}=152.93\times {\left(\text{Tmean}/\text{Bmin}\right)}^{‐0.27}$$

with a coefficient of determination $R^{2}=0.65$.

This function can be used as a “threshold adjustment function” iteratively for segmenting lesions on PET images that are characterized by the Tmean/Bmin parameters.

Regarding the values of %Tmean in terms of Tmax/Bmin obtained from the second group, the mathematical function of the chosen regression curve that represents the correlation between these parameters is (function #6):

(6) $$\%\text{Tmean}=151.62\times {\left(\text{Tmax}/\text{Bmin}\right)}^{‐0.24}$$

with a coefficient of determination $R^{2}=0.74$.

Regarding the values of Tmax in terms of Tmean obtained from the second group, the mathematical function of the linear regression curve that represents the correlation between these parameters is (function #7):

(7) Tmax=1.73×Tmean‐1903.8

with a coefficient of determination $R^{2}=0.96$.

By combining functions #6 and #7, function #8 is obtained:

(8) $$\%\text{Tmean}=151.62\times {\left[1.73\times (\text{Tmean}/\text{Bmin})‐1903.8/\text{Bmin}\right]}^{‐0.24}$$

with a coefficient of determination $R^{2}=0.99$. (This coefficient of determination is calculated manually by generating a mathematical function on Microsoft Excel.)

### C. Functions comparison

(Figure 2b) shows the difference between function #8 (obtained using histological measurements as a reference) and function #4 (obtained by using CT images as a reference and represented in (Fig. 2a) in three‐dimensions. (Figure 2c) shows the same difference listed in (Fig. 2b) but here with the locations of the “clinical points”. Each “clinical point” represents the location of a clinical lesion used for adjustment in this study. For a better distinction of each “clinical point” location on the plot area, the value of the difference is imposed to “‐3” for every “clinical point”. In some cases the “clinical points” are overlapping. For all “clinical points” (for 65 lesions), the maximum difference between the two functions is equal to 7.3% (“Z” value in (Fig. 2c).

### D. Application and validation of the segmentation technique

**Figure 3:**
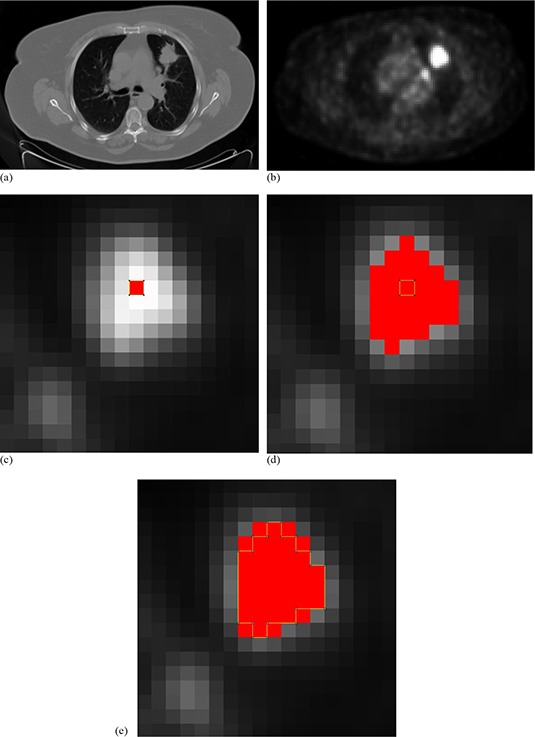
CT image (from FDG PET/CT exam) for lesion #62 (a). FDG PET image (Bmin=464) (b). Zoom on FDG PET image, maximum intensity in the lesion appears in red (Tmean=Tmax=22146), first iteration (c) — FDG lesion uptake in red (%Tmean=52.88% and Threshold=11711, that gives Tmean=17385); second iteration (d) — FDG lesion uptake in red (%Tmean=55.92% and Threshold=9722, that gives Tmean=16214). Convergence at the third iteration (e) (%Tmean=56.83% and Threshold=9215) to a constant lesion area on the PET image (Area $\text{PET}=62{\text{mm}}^{2}$).

Since the available histological data were used for the adjustment of function # 8 (of HG) this function cannot be validated using the same histological data. For this reason, the model from the CT data is chosen as the final model for validation. Therefore, function #4 (which was adjusted from CTG images) was validated. The validation was based only on histological measurements of the 27 lesions from the HG. Ten lesions with a MaAxis Hist smaller than or equal to 20 mm (Table 1), and 17 lesions with MaAxis Hist between 20 and 45 mm (Table 2) were found. For each measured lesion, MaAxis PET and Area PET are obtained using the “threshold adjustment function”, function #4. To better understand the automatic procedure of the algorithm, Fig. 3 shows an example of the iterations used for the segmentation of lesion #62 on its PET image. First, for the selected PET slice ((Fig. 3a), the corresponding CT slice ((Fig. 3b) was found by an image fusion software. Therefore, to automatically identify the lung region on the PET image, the corresponding CT image was superimposed after lung CT segmentation (by an appropriate software). In the identified lungs regions on the PET image, the minimum intensity Bmin was measured (Bmin=464). This value was inserted into Bmin's place in function #4. Bmin is a fixed parameter and remains constant for all iterations. For the first iteration, the value of Tmean (mean intensity in the lesion area in the PET image) in function #4 was imposed equal to the value of the maximum intensity Tmax (Tmean=Tmax=22146). For each iteration, the value of the Tmean parameter of the lesion in the PET image was measured. Then, that value was used in the threshold adjustment function (#4) in order to return a threshold value (in (Fig. 3c), Threshold=11711 for the first iteration). This threshold gives a new tumor size ((Fig. 3d) and, consequently, new specific parameter (Tmean) values. The iterations were stopped when the specific parameters stopped changing from one iteration to another ((Fig. 3e). Afterwards, MaAxis PET, MaAxis CT, and MaAxis Hist were compared, and then Area PET was compared to the measured Area CT on the corresponding CT image. Note that ΔVs MaAxis PET‐Hist is the relative difference between MaAxis PET and MaAxis Hist; ΔVs MaAxis PET‐CT is the relative difference between MaAxis PET and MaAxis CT; ΔVs MaAxis CT‐Hist is the relative difference between MaAxis CT and MaAxis Hist; and ΔVs Area PET‐CT is the relative difference between Area PET and Area CT.

**Table 1:** Validation of the segmentation technique for lesion having long axis smaller than 20 mm.

*Lesion #*	*MaAxis Hist (mm)*	*Bmin*	*Tmean*	*%Tmean*	*MaAxis PET (mm)*	*MaAxis CT (mm)*	*ΔVs MaAxis PET‐Hist (%)*	*ΔVs MaAxis CT‐Hist (%)*	*ΔVs MaAxis PET‐CT (%)*	*Area PET (mm* ^*2*^ *)*	*Area CT (mm* ^*2*^ *)*	*ΔVs Area PET‐CT (%)*	*COV*
39	12	560	6407.7	74.3	14.4	11.8	+20.0	*‐* 1.7	+22.0	96	87.9	+9.2	0.16
40	12	378	4081.7	77.1	17.9	12.7	+49.2	+5.8	+40.9	144	90.6	+58.9	0.16
41	12	374	2680.3	87.8	17.9	15.4	+49.2	+28.3	+16.2	144	122.2	+17.8	0.10
42	15	392	2939.0	86.0	14.4	17.9	−4.0	+19.3	−19.6	112	94.7	+18.3	0.05
43	16	151	2686.7	71.9	25.6	17.7	+60.0	+10.6	+44.6	304	152.4	+99.5	0.23
44	18	360	4098.0	76.2	17.0	16.3	−5.6	−9.4	+4.3	128	144.2	−11.2	0.15
45	18	350	7216.0	65.0	20.0	18.5	+11.1	+2.8	+8.1	208	182.6	+13.9	0.25
46	19	179	5218.0	61.1	20.0	19.3	+5.3	+1.6	+3.6	244	162.0	+50.6	0.31
47	20	195	1949.3	86.2	25.6	16.7	+28.0	−16.5	+53.3	244	159.3	+53.2	0.10
48	20	467	4571.0	78.2	26.8	23.7	+34.0	+18.5	+13.1	400	344.7	+16.0	0.15
Average:	16.2	340.6	4184.8	76.4	19.96	17.0	+24.7	+5.9	+18.7	202.4	149.5	+32.6	0.17
Standard deviation:	±3.1	±123.7	±1625.5	±8.4	±4.4	±3.4	±22.1	±13.7	±22.2	±92.0	±78.5	±31.7	±0.08

**Table 2:** Validation of the segmentation technique for lesion having long axis between 20 and 45 mm.

*Lesion #*	*MaAxis Hist (mm)*	*Bmin*	*Tmean*	*%Tmean*	*MaAxis PET (mm)*	*MaAxis CT (mm)*	*ΔVs MaAxis PET‐Hist (%)*	*ΔVs MaAxis CT‐Hist (%)*	*ΔVs MaAxis PET‐CT (%)*	*Area PET (mm* ^*2*^ *)*	*Area CT (mm* ^*2*^ *)*	*ΔVs Area PET‐CT (%)*	*COV*
49	22	279	3566.6	75.0	20.0	18.1	−9.1	−17.7	+10.5	208	160.7	+29.4	0.22
50	24	278	4161.0	71.7	20.0	23.4	−16.7	−2.5	−14.5	144	255.4	−43.6	0.16
51	25	167	5040.3	60.7	28.8	17.6	+15.2	−29.6	+63.6	384	184.0	+108.7	0.30
52	25	193	9476.0	53.3	26.8	23.7	+7.2	−5.2	+13.1	384	296.6	+29.5	0,36
53	25	263	10442.9	55.7	25.6	24.2	+2.4	−3.2	+5.8	352	328.2	+7.3	0.32
54	25	335	7954.2	62.8	25.6	23.7	+2.4	−5.2	+8.0	352	332.3	+5.9	0.23
55	25	295	7441.4	62.1	28.8	25.3	+15.2	+1.2	+13.8	384	392.8	−2.2	0.26
56	28	191	11198.8	51.1	28.8	23.4	+2.9	−16.4	+23.1	400	309.0	+29.4	0.37
57	28	776	16033.8	63.8	26.8	33.6	−4.3	+20.0	−20.2	416	589.1	−29.4	0.23
58	28	632	7253.0	73.9	28.8	36.5	+2.9	+30.4	−21.1	416	604.2	−31.1	0.20
59	32	541	13026.3	61.9	26.8	25.7	−16.3	−19.7	+4.3	416	414.7	+0.3	0.28
60	32	316	19113.9	50.3	32.2	33.0	+0.6	+3.1	−2.4	592	605.6	−2.2	0.34
61	35	202	15326.0	48.0	32.2	29.5	−8.0	−15.7	+9.2	544	450.0	+20.9	0.35
62	35	464	16213.8	56.8	35.7	33.8	+2.0	−3.4	+5.6	624	672.9	−7.3	0.24
63	40	394	11967.8	58.9	41.8	42.8	+4.5	+7.0	−2.3	1056	1064.3	−0.8	0.24
64	42	464	5558.0	74.0	37.7	40.4	−10.2	−3.8	−6.7	736	979.2	−24.8	0.15
65	45	406	13169.8	58.0	43.3	39.7	−3.8	−11.8	+9.1	848	850.1	−0.2	0.31
Average:	30.4	364.5	10408.5	61.1	+30.0	29.1	−0.8	−4.3	+5.8	485.6	499.4	+5.3	0.27
Standard deviation:	±6.7	±163.2	±4515.9	±8.3	±6.4	±7.8	±9.0	±14.6	±19.2	±221.7	±261.4	±33.3	±0.07

As presented in Table 1, for lesions with a major axis smaller than or equal to 20 mm, ΔVs MaAxis PET‐Hist values vary between −4.0% and +60% (average +24.7%, standard deviation ±22.1), ΔVs MaAxis PET‐CT values vary between −19.6% and +53.3% (average +18.7%, standard deviation ±22.2), ΔVs MaAxis CT‐Hist values vary between −16.5% and +28.3% (average +5.9%, standard deviation ±13.7), and ΔVs Area PET‐CT values vary between −11.2% and +99.5% (average +32.6%, standard deviation ±31.7). From Table 2, for lesions with a major axis ranging between 20 and 45 mm, ΔVs MaAxis PET‐Hist values vary between −16.7% and +15.2% (average −0.8%, standard deviation ±9.0), ΔVs MaAxis PET‐CT values vary between −21.1% and +63.6% (average +5.8%, standard deviation ±19.2), ΔVs MaAxis CT‐Hist values vary between −29.6% and +30.4% (average −4.3%, standard deviation ±14.6), and ΔVs Area PET‐CT values vary between −43.6% and +108.7% (average +5.3%, standard deviation ±33.3).

**Figure 4:**
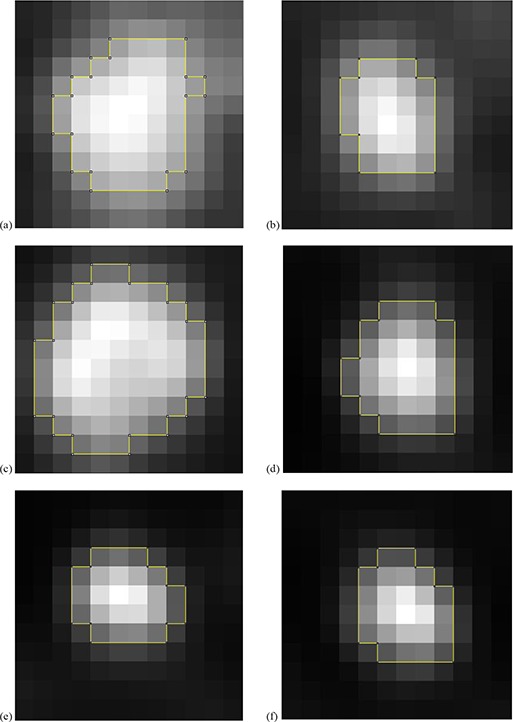
Heterogeneity estimation and Area PET (outlined in yellow) for 6 tumors: COV=0.15 for lesion #64 (a); COV=0.20 for lesion #58 (b); COV=0.24 for lesion #63 (c); COV=0.35 for lesion #61 (d); COV=0.36 for lesion #52 (e); COV=0.37 for lesion #56 (f).

In addition, in Tables 1and 2, the ^18^F‐FDG uptake heterogeneity was estimated using the coefficient of variation (COV), defined as the ratio between the SD (standard deviation) of the standardized uptake values and the mean standardized uptake value within the delineated PET image. For lesions with a major axis smaller than or equal to 20 mm (Table 1), COV values vary between 0.05 and 0.31 (average 0.17, standard deviation ±0.08). For lesions with a major axis ranging between 20 and 45 mm, COV values vary between 0.15 and 0.37 (average 0.27, standard deviation ±0.07). Figure 4 shows the delineation of different lesions and their associated COV.

## IV. DISCUSSION

Tumor delineation is a very important step in radiotherapy treatment planning. Recently, for non‐small cell lung cancers (NSCLC), in addition to using CT images, it has been recommended to use functional PET images for tumor segmentation, to take into account the functional characteristics of the target.^2^, ^8^ Until now, there was no satisfactory automatic PET image segmentation technique for clinical application.

The present study shows a new strategy for automatic segmentation on PET images by using adaptive thresholding techniques.^12^, ^16^ For more precision, this strategy consists of adjusting threshold functions directly from the information extracted from the patient's body, rather than from phantoms. Two references are used to obtain the threshold adjustment function. The first reference is based on the CT lesion images, and the second on the histological measurements of the surgically removed lesions.

Functions #4 and #8, adjusted respectively from CT images and histological measurements as references, were compared. In (Fig. 2c), the difference between functions #4 and #8 was smaller than or equal to 7.3% for all 65 lesions used for adjustment in this study (“clinical points”). Therefore, the use of one of these two references is sufficient to obtain a “threshold adjustment function”. As a result, only function #4 adjusted from CT images was validated. This difference could be decreased and the technique's precision increased if: 1) the measurement technique on surgical specimens for HG is improved; or 2) the CT images are acquired with optimal acquisition parameters (e.g., the slice thickness or/and the space between slice images is less than 5 mm) for the CTG. Also, by using a PET image pixel size less than $4\;\times \;4\;{\text{mm}}^{2}$, partial volume effects (PVE) could be reduced.

Function #4 was obtained using CTG lesions (38 lesions) and was used to validate the present segmentation strategy. It was thereby applied to segment the other 27 lesions (lesions of HG). The resulting lesions' sizes were compared to the results obtained from their corresponding CT images and to histological data. The 27 HG lesions were separated into two groups according to their sizes. The obtained lesions' sizes, with axes in the range of 20 to 45 mm, provided good similarities with the histological measurements (mean ΔVs MaAxis PET−Hist=−0.8%±9.0%) and an acceptable similarity with the CT measurements (mean ΔVs Area PET−CT=+5.3%±33.3% and mean ΔVs MaAxis PET−CT=+5.8%±19.2%). The present technique showed great accuracy in PET/CT image segmentation and therefore is effective for segmentation of lung cancer lesions with different activities and variable image parameters. It is interesting to compare our results with those published recently by Hatt et al.^(21)^ In this study, the authors tested different delineation methods on 17 NSCLCs. They compared the major axis on segmented PET images (by using different segmentation methods) to the major axis found by macroscopic histological measurements. By using a classic adaptive threshold technique proposed by Nestle et al.,^(13)^ Hatt et al. found for two observers: mean relative difference between the PET major axis and the histological major axis (mean ΔVs MaAxis PET‐Hist in our study) equal to ‐11% ±17% for the first observer, and equal to ‐12% ±16% for the second observer. It was necessary to choose two observers because the Nestle et al. method requires the definition of a manual background region of interest in the lungs, at a distance of several centimeters from the boundaries of the tumors. This is not the case in our strategy, because Bmin (representing the minimum intensity measured in the two lungs, on the PET image) is automatically determined by a mask removed from the CT image, to define lung regions on the PET image. Thus, our method had the best repeatability because it gives the same result when applied multiple times to the same image. In addition, a significant difference between adaptive thresholding results of the works of Hatt et al. and our results confirm the improvement of accuracy introduced by our strategy to the adaptive thresholding method. However, the same authors attributed the most accurate results to the Fuzzy Locally Adaptive Bayesian (FLAB) algorithm.^(25)^ They have observed a mean relative difference between the PET major axis and the histological major axis (mean ΔVs MaAxis PET‐Hist in our study) equal to ‐4% ±8%. Therefore, our new strategy for automatic tumor segmentation by adaptive thresholding provided the best estimation of the tumor major axis (−0.8%±9.0%), in discordance with many studies on classic adaptive thresholding segmentation. Finally, Hatt et al.^(21)^ identified in their study that for a case of large, heterogeneous NSCLCs, adaptive thresholding should not be used for the delineation of ^18^F‐FDG PET uptake. But in our study, the iterative use of Tmean (mean intensities (gray levels) forming tumor area on PET image) allows us to take into account, indirectly and partially, tumor heterogeneity. In addition, to be more accurate for large tumor, it is also possible in later studies to represent tumor heterogeneity and volume by parameters in the threshold adjustment function.

**Figure 5:**
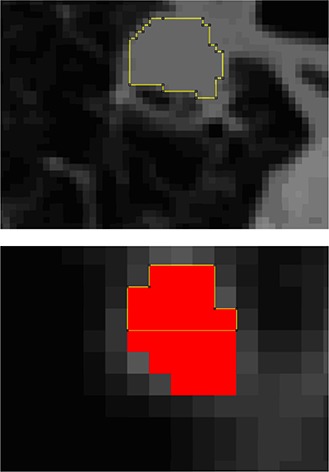
Segmentation of lesion #51: on CT image (a), and on the FDG PET image (in red) (b) where the segment outlined in yellow represents the area segmented on CT image (a).

Lesion #51 (Fig. 5) showed an underestimation of the lesion area on the CT image in relation to the lesion area on the PET image (ΔVs Area PET−CT=+108.7% and ΔVs MaAxis PET−CT=+63.6%). This is not the case for the comparison of the lesion size of the PET image with the lesion size of histological measurements (ΔVs MaAxis PET‐Hist =+15.2%). Indeed, the lesion presented on the CT image had a very heterogeneous lower segment that was not considered in the segmentation (yellow lines in (Fig. 5a). Histological data confirm the presence of microscopic extensions in this segment of the lesion. The extensions were represented by a faint FDG uptake and thus were correctly segmented on the PET image (the lower segment of red area in (Fig. 5b). This example emphasizes the usefulness of lesion segmentation on PET images with the present strategy.

For lesions with a major axis smaller than or equal to 20 mm, an overestimation of the measurements derived from histological or CT data was found. The probable reasons for this deficiency are the residue of the respiratory movements and/or the PVE, which reflects the impact of tumor size relative to the system spatial resolution. It is obvious that, depending on the tumor uptake, the lesion will either be undetected or overestimated.

This segmentation strategy is simple and easily implementable in the clinic. In addition, it is possible to create an algorithm that could automatically adjust the threshold function for different PET/CT systems after the selection of a set of lesions (used as a reference) by an expert.

One of the main difficulties limiting the segmentation of lung tumors by PET/CT images is the noise due to the patient's respiratory movements. Many studies have taken into account these movements that disrupt PET images.^(26)^ For this reason, the present study considers only lesions located in the upper lobe of the lungs where movements are less significant.^23^, ^24^ The new generation of PET/CT devices, characterized by improved accuracy, will give more precision, allowing for the application of this technique to tumors of various sizes, and to those located also in lower and middle lobes of the lungs. With such devices, it will also be possible to take into account different respiratory cycle times within different “threshold adjustment functions”, without using respiratory gating methods.

In this study, only the macroscopic histological measurements were used as references for adjustment and validation of the threshold function. Therefore, the microscopic spread was not measured and the tumor was underestimated on histological measurements. The microscopic histological reports, the degree of tumor necrosis, and the importance of pulmonary diseases reaction were only used for lesions selection. In addition, deformations of lungs must also be better considered in histological measurements. Note that it is indeed possible to obtain more accurate measurements and significance by taking into account the microscopic spread, the entire histological volume (delineations on three dimensions), and better consideration of lung deformations through a manner similar to studies already cited.^8^, ^19^, ^22^

## V. CONCLUSIONS

The originality of this study is the use of *in vivo* data for the development of a segmentation algorithm for lung tumors on PET images. Two different references were used: the first deduced from CT images, and the second from histological measurements. The resulting algorithm showed good accuracy for lesions with a major axis greater than 20 mm. Therefore, this new strategy reveals an important solution for the segmentation problem on PET images. However, this algorithm presented inaccuracies for the segmentation of small size lesions. The problem could be mainly due to the partial volume effects related to the resolution of the PET/CT scan and/or to the respiratory motions. In order to increase the measurements' accuracy, further studies should take into account respiratory movements (using new and more accurate PET/CT devices), lesion sizes, and three‐dimensional microscopic measurements of lesions. In conclusion, for PET image segmentation, adaptive thresholding approaches can produce good results, provided that a suitable strategy is used.

## ACKNOWLEDGMENTS

The authors wish to thank the Pôle d'Activitée Médicale en imagerie of the Hospices Civils de Lyon and the Centre Hospitalier du Nord of Lebanon for the financial support.
